# Epidemiology of Cancer-Associated Venous Thromboembolism in Patients With Solid and Hematologic Neoplasms in the Veterans Affairs Health Care System

**DOI:** 10.1001/jamanetworkopen.2023.17945

**Published:** 2023-06-12

**Authors:** Kylee L. Martens, Ang Li, Jennifer La, Sarah B. May, Kaitlin N. Swinnerton, Hannah Tosi, Danne C. Elbers, Nhan V. Do, Mary T. Brophy, J. Michael Gaziano, Saran Lotfollahzadeh, Vipul Chitalia, Katya Ravid, Nathanael R. Fillmore

**Affiliations:** 1 Division of Hematology & Medical Oncology, Oregon Health & Science University, Portland; 2 Section of Hematology-Oncology, Baylor College of Medicine, Houston, Texas; 3 Massachusetts Veterans Epidemiology Research and Information Center, VA Boston Healthcare System, Boston; 4 Department of Medicine, Harvard Medical School, Boston, Massachusetts; 5 Institute for Clinical & Translational Research, Baylor College of Medicine, Houston, Texas; 6 Boston University School of Medicine, Boston, Massachusetts

## Abstract

**Importance:**

Identifying changes in epidemiologic patterns of the incidence and risk of cancer-associated thrombosis (CAT), particularly with evolving cancer-directed therapy, is essential for risk stratification.

**Objective:**

To assess the incidence of CAT over time and to determine pertinent patient-specific, cancer-specific, and treatment-specific factors associated with its risk.

**Design, Setting, and Participants:**

This longitudinal, retrospective cohort study was conducted from 2006 to 2021. Duration of follow-up was from the date of diagnosis until first venous thromboembolism (VTE) event, death, loss of follow-up (defined as a 90-day gap without clinical encounters), or administrative censoring on April 1, 2022. The study took place within the US Department of Veterans Affairs national health care system. Patients with newly diagnosed invasive solid tumors and hematologic neoplasms were included in the study. Data were analyzed from December 2022 to February 2023.

**Exposure:**

Newly diagnosed invasive solid tumors and hematologic neoplasms.

**Main Outcomes:**

Incidence of VTE was assessed using a combination of *International Classification of Diseases, Ninth Revision, Clinical Modification* and *International Statistical Classification of Diseases, Tenth Revision, Clinical Modification *and natural language processing confirmed outcomes. Cumulative incidence competing risk functions were used to estimate incidence of CAT. Multivariable Cox regression models were built to assess the association of baseline variables with CAT. Pertinent patient variables included demographics, region, rurality, area deprivation index, National Cancer Institute comorbidity index, cancer type, staging, first-line systemic treatment within 3 months (time-varying covariate), and other factors that could be associated with the risk of VTE.

**Results:**

A total of 434 203 patients (420 244 men [96.8%]; median [IQR] age, 67 [62-74] years; 7414 Asian or Pacific Islander patients [1.7%]; 20 193 Hispanic patients [4.7%]; 89 371 non-Hispanic Black patients [20.6%]; 313 157 non-Hispanic White patients [72.1%]) met the inclusion criteria. Overall incidence of CAT at 12 months was 4.5%, with yearly trends ranging stably from 4.2% to 4.7%. The risk of VTE was associated with cancer type and stage. In addition to confirming well-known risk distribution among patients with solid tumors, a higher risk of VTE was observed among patients with aggressive lymphoid neoplasms compared with patients with indolent lymphoid or myeloid hematologic neoplasms. Compared with no treatment, patients receiving first-line chemotherapy (hazard ratio [HR], 1.44; 95% CI, 1.40-1.49) and immune checkpoint inhibitors (HR, 1.49; 95% CI, 1.22-1.82) had a higher adjusted relative risk than patients receiving targeted therapy (HR, 1.21; 95% CI, 1.13-1.30) or endocrine therapy (HR, 1.20; 95% CI, 1.12-1.28). Finally, adjusted VTE risk was significantly higher among Non-Hispanic Black patients (HR, 1.23; 95% CI, 1.19-1.27) and significantly lower in Asian or Pacific Islander patients (HR, 0.84; 95% CI, 0.76-0.93) compared with Non-Hispanic White patients.

**Conclusions and Relevance:**

In this cohort study of patients with cancer, a high incidence of VTE was observed, with yearly trends that remained stable over the 16-year study period. Both novel and known factors associated with the risk of CAT were identified, providing valuable and applicable insights in this current treatment landscape.

## Key Points

**Question:**

Have patterns of the incidence and risk of cancer-associated thrombosis (CAT) changed over time with the evolution of cancer-directed therapy?

**Findings:**

In this cohort study of 434 203 patients with solid tumor and hematologic neoplasms in the Department of Veterans Affairs health care system, a high incidence of CAT with yearly trends ranging stably from 4.2% to 4.7% over the 16-year study period was observed. Cancer type and stage and systemic treatment were significantly associated with CAT, and patients of different races and ethnicities demonstrated varying degrees of risk of CAT.

**Meaning:**

These findings suggest that patient-specific and treatment-specific factors, accounting for changes in the treatment landscape over time, warrant consideration in future venous thromboembolism risk stratification models.

## Teaser

This cohort study assesses the incidence of cancer-associated venous thromboemoblism (VTE) among patients in the Department of Veterans Affairs health care system with solid and hematologic neoplasms and analyzes the patient-specific, cancer-specific, and treatment-specific factors associated with the risk of VTE.

## Introduction

Venous thromboembolism (VTE) is 1 of the leading causes of non–cancer-related mortality in patients with active cancer receiving systemic therapy.^1^ Patients with cancer are not only at increased risk of VTE compared with the general population, but also at higher risk of VTE recurrence despite receipt of anticoagulation therapy.^2,3,4,5^ Our understanding of the pathophysiology of cancer-associated thrombosis (CAT) has evolved over the past few decades, as have validated tools to identify those at high risk.^6,7,8^ Despite these advances, there remains a critical need to identify the associations of race and ethnicity, cancer subtype, and systemic therapy regimens with CAT, particularly over time as the landscape of antineoplastic therapies has evolved.

Recent large retrospective cohort studies^9,10^ that relied on claims diagnosis codes reported higher incidence of CAT over time within California and Denmark. However, major changes in the *International Classification of Diseases, Ninth Revision, Clinical Modification (ICD-9-CM) and International Statistical Classification of Diseases, Tenth Revision, Clinical Modification (ICD-10-CM) *codes for deep venous thrombosis (DVT) occurred in 2004, 2009, and 2015, thereby making comparisons of VTE incidence challenging to interpret. Changes in systemic therapy regimens, particularly with the introduction of immunotherapy and targeted therapy into the first-line setting, have led to several studies evaluating VTE risk compared with standard chemotherapy.^11,12^ Additionally, recent retrospective analyses^13,14^ exploring patterns of CAT have identified discrepant incidence and risk among patients of different races and ethnicities; however, limitations of these prior studies^13,14^ include geographic restrictions (California and Texas), thereby limiting generalizability of trends among the US population. Greater emphasis on identifying patterns of CAT incidence and risk, incorporating patient-specific, cancer-specific, and treatment-specific factors in a racially, ethnically, and geographically diverse population is of critical importance for optimal treatment and prognostic assessment.

Using integrated data from the US Department of Veterans Affairs (VA) national health care system, we conducted a longitudinal cohort analysis of patients with cancer in the US to characterize the incidence, associated factors, and outcomes of CAT. We specifically assessed the pattern of VTE incidence over time, as well as the association of cancer type, treatment, race and ethnicity, US region, and other relevant factors, with the occurrence of CAT and overall survival in this patient population.

## Methods

### Study Population

This longitudinal cohort study was approved by the VA Boston Research and Development committee as an exempt study prior to data collection and analysis with a waiver of informed consent due to the use of existing data, per the Common Rule. It followed the Strengthening the Reporting of Observational Studies in Epidemiology (STROBE) reporting guideline for cohort studies. We performed a retrospective analysis of patients with newly diagnosed invasive cancer at the VA health care system from 2006 to 2021. Patients with an incident cancer diagnosis were identified using national VA cancer registry data and were linked to electronic health record data in the VA Corporate Data Warehouse. Linkage, data harmonization, cohort exclusion, and variable extraction are described in detail in eMethods in Supplement 1. Index date was defined as the date of histologic cancer diagnosis. Patients were excluded if they had a benign histologic diagnosis, cancer in-situ, or stage 0 cancer diagnosis; if the diagnosis was nonmelanomatous skin cancer; if they were not a primary VA user; or if they had historical VTE within 6 months prior to their cancer diagnosis (eFigure 1 in Supplement 1). For CAT analysis, patients were followed from the date of diagnosis until the first VTE event, death, loss of follow-up (defined as a 90-day gap without clinical encounters), or administrative censoring on April 1, 2022.

### Baseline Characteristics

Baseline demographic data, including age, sex, self-reported race and ethnicity (defined as Hispanic, non-Hispanic Asian or Pacific Islander, non-Hispanic Black, or non-Hispanic White), US region, and rurality, were tabulated. Race and ethnicity were included to assess the association with CAT. To determine socioeconomic status, the Area Deprivation Index (ADI) score was calculated as described in the eMethods in Supplement 1.^15,16^ Pertinent medical history and medications, *ICD-9-CM* and *ICD-10-CM* diagnosis and procedure codes, National Cancer Institute comorbidity index (NCI-CI),^17^ body mass index (BMI; calculated as weight in kilograms divided by height in meters squared), lifetime history of VTE, recent prolonged hospitalization (more than 3 days within last 3 months), history of paralysis, baseline laboratory values (white blood cell count, hemoglobin, and platelet count), anticoagulant or antiplatelet use (obtained through outpatient pharmacy records within last 3 months), and overall mortality were also obtained. Cancer-specific information included date of diagnosis, cancer histologic profile, American Joint Committee on Cancer staging, and time delay between diagnosis and treatment. First-line systemic therapy regimen within the first 3 months of cancer diagnosis was defined as a time-varying categorical variable using all concurrently administered initial therapies, divided into mutually exclusive categories including chemotherapy (with or without immunotherapy, targeted, or endocrine), PD-1 (programmed cell death protein 1) and PD-L1 (programmed death-ligand 1) immune checkpoint inhibitor (with or without targeted or endocrine therapy), targeted therapy (with or without endocrine therapy), and endocrine monotherapy. Systemic therapy classifications can be found in eTable 1 in Supplement 1.

### Outcome Definitions

The primary composite outcome of first VTE was defined as radiologically confirmed symptomatic or asymptomatic pulmonary embolism (PE), proximal or distal lower extremity DVT (LE-DVT), and upper-extremity, catheter-related DVT (CR-DVT). Tumor thrombus, splanchnic vein, or cerebral venous sinus thromboses were excluded to ensure uniform outcome assessment and interpretation. A thrombotic event meeting criteria for CAT was an event that occurred at any time after index date of cancer diagnosis up to 12 months to limit time-varying confounding from relapsed or advanced disease. VTE events were screened using a combination of *ICD-9-CM and ICD-10-CM* codes and natural language processing radiology impressions from inpatient and outpatient settings, as described previously.^18^ This combined algorithm had a sensitivity of 96% and a positive predictive value of 91% when compared with manual medical record abstraction in the VA cohort as shown previously.^8^

### Statistical Analysis

Descriptive statistics were used to examine the distribution of categorical and continuous variables. Stratified cumulative incidence curves were used to estimate incidence of VTE while accounting for early death as a competing risk. Multivariable cause-specific Cox regression models were created to assess factors associated with the risk of overall VTE, PE, or LE-DVT, and mortality. Baseline variables were chosen on the basis of prior clinical knowledge of prothrombotic factors and included age per 10-year increase, sex, race and ethnicity, US region, rurality, ADI score, NCI-CI, diagnosis year, cancer type and histologic category, cancer stage, history of VTE at least 6 months prior to cancer diagnosis, history of paralysis, recent prolonged hospitalization, baseline antiplatelet or anticoagulant use, and Khorana score components (BMI ≥35, white blood cell count >11 000 cells/μL [to convert to cells × 10^9^/L, multiply by 0.001], hemoglobin <10 g/dL [to convert to grams per liter, multiply by 10], and platelet count ≥350 × 10^3^/μL [to convert to × 10^9^/L, multiply by 1]).^6^ First-line systemic treatment regimen within 3 months was included in the analysis as a time-varying covariate. All statistical analyses were performed using Stata statistical software version 17 (StataCorp). Data were analyzed from December 2022 to February 2023.

## Results

### Cohort Characteristics

From 2006 to 2021, 434 203 patients (420 244 men [96.8%]; median [IQR] age 67 [62-74] years) with newly diagnosed invasive cancer in the VA health care system met inclusion criteria (eFigure 1 in Supplement 1). Table 1 summarizes baseline characteristics of the overall cohort, stratified by race and ethnicity. Most patients self-identified as non-Hispanic White (313 157 patients [72.1%]), with 89 371 patients (20.6%) identifying as non-Hispanic Black, 20 193 patients (4.7%) as Hispanic, and 7414 patients (1.7%) as Asian or Pacific Islander. The most common cancer types were prostate cancer (133 085 patients [30.7%]), lung cancer (75 441 patients [17.4%]), colon cancer (29 580 patients [6.8%]), and head and neck cancer (25 486 patients [5.9%]); a total of 162 666 patients (37.5%) had stage III or IV (advanced) cancer at diagnosis. Among the 118 731 patients (27.3%) who received systemic therapy within the first 90 days of diagnosis, the median (IQR) time delay from diagnosis to first treatment was 37 (20-57) days.

**Table 1.** Patient Characteristics Stratified by Race and Ethnicity^a^

Variable	Patients, No. (%)				
	Overall (N = 434 203)	Hispanic (n = 20 193)	Non-Hispanic Asian or Pacific Islander (n = 7414)	Non-Hispanic Black (n = 89 371)	Non-Hispanic White (n = 313 157)
Age, median (IQR), y	67 (62-74)	68 (61-75)	67 (61-73)	64 (59-70)	68 (63-74)
Sex					
Male	420 244 (96.8)	19 625 (98.2)	7033 (94.9)	85 988 (96.2)	303 674 (97.0)
Female	13 959 (3.2)	568 (2.8)	381 (5.1)	3383 (3.8)	9483 (3.0)
Rurality					
Urban	278 015 (64.0)	15 990 (79.2)	4946 (66.7)	74 272 (83.1)	180 353 (57.6)
Rural	152 086 (35.0)	2946 (14.6)	2390 (32.2)	14 570 (16.3)	130 599 (41.7)
Unknown	4102 (0.9)	1257 (6.2)	78 (1.1)	529 (0.6)	2205 (0.7)
US region					
Southeast	87 198 (20.1)	7542 (37.3)	988 (13.3)	20 973 (23.5)	57 078 (18.2)
Continental	75 566 (17.4)	3951 (19.6)	1411 (19.0)	17 722 (19.8)	51 940 (16.6)
Midwest	100 393 (23.1)	1060 (5.2)	1353 (18.2)	17 221 (19.3)	79 781 (25.5)
North Atlantic	94 517 (21.8)	1978 (9.8)	868 (11.7)	24 638 (27.6)	66 637 (21.3)
Pacific	76 529 (17.6)	5662 (28.0)	2794 (37.7)	8817 (9.9)	57 721 (18.4)
National Area Deprivation Index, percentile					
0-25	53 709 (12.4)	3126 (15.5)	1605 (21.6)	9916 (11.1)	38 397 (12.3)
26-50	102 745 (23.7)	3931 (19.5)	1786 (24.1)	16 646 (18.6)	79 298 (25.3)
51-75	128 681 (29.6)	22 193 (24.8)	1815 (24.5)	22 193 (24.8)	99 268 (31.7)
76-100	140 391 (32.3)	8543 (42.3)	2032 (27.4)	37 121 (41.5)	91 595 (29.2)
Unknown	8677 (2.0)	326 (1.6)	176 (2.4)	3495 (3.9)	4599 (1.5)
Baseline comorbidities					
National Cancer Institute Comorbidity Index, median (IQR)	0.29 (0.00-0.58)	0.29 (0.00-0.72)	0.29 (0.00-0.57)	0.29 (0.00-0.64)	0.29 (0.00-0.57)
Body mass index ≥35^b^	72 158 (16.6)	2967 (14.7)	1204 (16.2)	53 314 (17.0)	53 314 (17.0)
History of venous thromboembolism	18 154 (4.2)	738 (3.7)	253 (3.4)	3714 (4.2)	13 290 (4.2)
History of paralysis	3313 (0.8)	152 (0.8)	55 (0.7)	857 (1.0)	2220 (0.7)
Recent hospitalization (90 d)	57 023 (13.1)	3068 (15.2)	945 (12.7)	12 283 (13.7)	40 238 (12.8)
Baseline anticoagulant					
Warfarin	17 010 (3.9)	504 (2.5)	252 (3.4)	1841 (2.1)	14 281 (4.6)
Low-molecular-weight heparin	3404 (0.8)	170 (0.8)	45 (0.6)	515 (0.6)	2655 (0.8)
Direct oral anticoagulant	6593 (1.5)	187 (0.9)	102 (1.4)	5463 (1.7)	5463 (1.7)
Baseline antiplatelet					
Aspirin	68 250 (15.7)	3952 (19.6)	1309 (17.7)	19 417 (21.7)	43 028 (13.7)
P2Y12 inhibitor^c^	18 412 (4.2)	760 (3.8)	327 (4.4)	2239 (2.5)	14 929 (4.8)
Aspirin and P2Y12^c^ inhibitor	1522 (0.4)	71 (0.4)	25 (0.3)	320 (0.4)	1095 (0.3)
Other	2590 (0.6)	84 (0.4)	39 (0.5)	469 (0.5)	1979 (0.6)
Cancer type					
Prostate	133 085 (30.7)	6952 (34.4)	2221 (30.0)	40 853 (45.7)	81 932 (26.2)
Lung	75 441 (17.4)	1704 (8.4)	1112 (15.0)	12 054 (13.5)	59 860 (19.1)
Colon	29 580 (6.8)	1791 (8.9)	611 (8.2)	5388 (6.0)	21 505 (6.9)
Head and neck	25 486 (5.9)	962 (4.8)	377 (5.1)	3885 (4.3)	20 006 (6.4)
Kidney	18 315 (4.2)	1157 (5.7)	382 (5.2)	3700 (4.1)	12 898 (4.1)
Liver	17 406 (4.0)	1588 (7.9)	381 (5.1)	4281 (4.8)	10 984 (3.5)
Melanoma	16 702 (3.8)	194 (1.0)	168 (2.3)	119 (0.1)	16 094 (5.1)
Bladder	15 040 (3.5)	517 (2.6)	190 (2.6)	1416 (1.6)	12 779 (4.1)
Miscellaneous solid	13 763 (3.2)	664 (3.3)	228 (3.1)	2226 (2.5)	10 516 (3.4)
Gastric and esophageal	13 399 (3.1)	700 (3.5)	241 (3.3)	2238 (2.5)	10 074 (3.2)
Indolent non-Hodgkin lymphoma	8987 (2.1)	467 (2.3)	152 (2.1)	1134 (1.3)	7152 (2.3)
Pancreas	8925 (2.1)	417 (2.1)	166 (2.2)	1720 (1.9)	6508 (2.1)
Neuroendocrine	6831 (1.6)	355 (1.8)	113 (1.5)	1519 (1.7)	4779 (1.5)
Chronic lymphocytic leukemia	6486 (1.5)	197 (1.0)	88 (1.2)	743 (0.8)	5396 (1.7)
Multiple myeloma	6470 (1.5)	344 (1.7)	344 (1.7)	2014 (2.3)	3903 (1.2)
Breast	5075 (1.2)	241 (1.2)	145 (2.0)	1411 (1.6)	5075 (1.2)
Aggressive non-Hodgkin lymphoma	5351 (1.2)	343 (1.7)	127 (1.7)	715 (0.8)	4113 (1.3)
Myelodysplastic syndrome	4897 (1.1)	206 (1.0)	75 (1.0)	532 (0.6)	4041 (1.3)
Thyroid	4845 (1.1)	361 (1.8)	152 (2.1)	693 (0.8)	3581 (1.1)
Chronic myeloid leukemia	3840 (0.9)	177 (0.9)	68 (0.9)	614 (0.7)	2944 (0.9)
Sarcoma	3017 (0.7)	175 (0.9)	59 (0.8)	516 (0.6)	2241 (0.7)
Brain	2485 (0.6)	119 (0.6)	42 (0.6)	274 (0.3)	2019 (0.6)
Bile and gallbladder	2546 (0.6)	195 (1.0)	47 (0.6)	455 (0.5)	1819 (0.6)
Acute myeloid leukemia	2657 (0.6)	114 (0.6)	53 (0.7)	334 (0.4)	2130 (0.7)
Hodgkin lymphoma	1129 (0.3)	77 (0.4)	23 (0.3)	208 (0.2)	809 (0.3)
Gynecologic	1246 (0.3)	56 (0.3)	39 (0.5)	248 (0.3)	888 (0.3)
Testicular	1006 (0.2)	105 (0.5)	22 (0.3)	64 (0.1)	807 (0.3)
Acute lymphoblastic leukemia	193 (0.0)	15 (0.1)	0 (0.0)	17 (0.0)	154 (0.0)
Cancer stage					
I	107 814 (24.8)	5070 (25.1)	1831 (24.7)	19 868 (22.2)	80 112 (25.6)
II	122 020 (28.1)	6308 (31.2)	2041 (27.5)	32 519 (36.4)	80 149 (25.6)
III	50 754 (11.7)	2304 (11.4)	915 (12.3)	9865 (11.0)	37 181 (11.9)
IV	111 912 (25.8)	4657 (23.1)	1916 (25.8)	19 831 (22.2)	84 350 (26.9)
Unknown	41 703 (9.6)	1854 (9.2)	711 (9.6)	7288 (8.2)	31 365 (10.0)
First-line systemic therapy within 3 mo^d^					
None	315 472 (72.7)	14 751 (73.1)	5284 (71.3)	66 326 (74.2)	226 150 (72.2)
Chemotherapy	80 537 (18.5)	3445 (17.1)	1442 (19.4)	12 788 (14.3)	62 116 (19.8)
Immune checkpoint inhibitor	981 (0.2)	37 (0.2)	20 (0.3)	138 (0.2)	766 (0.2)
Targeted therapy	12 003 (2.8)	678 (3.4)	259 (3.5)	2622 (2.9)	8320 (2.7)
Endocrine therapy	25 210 (5.8)	1282 (6.3)	409 (5.5)	7497 (8.4)	15 805 (5.0)
Time to treatment initiation, median (IQR), d^e^	37 (20-57)	38 (20-59)	38 (20-58)	40 (21-60)	36 (20-56)

^a^ A total of 4068 patients with other or unknown race or ethnicity were excluded in the table.

^b^ BMI was calculated as weight in kilograms divided by height in meters squared.

^c^ P2Y12 inhibitors refer to clopidogrel, prasugrel, ticagrelor, and ticlopidine.

^d^ First-line systemic therapy was defined in mutually exclusive categories: Chemotherapy included chemotherapy and/or other concurrent therapy; immune checkpoint inhibitor included immune checkpoint inhibitor therapy and/or other concurrent therapy (excluding chemotherapy); targeted therapy included targeted therapy and/or other concurrent therapy (excluding chemotherapy and immune checkpoint inhibitor therapy); endocrine therapy included only endocrine therapy.

^e^ Time to treatment initiation was calculated from the subset of patients who received systemic therapy within 90 days after diagnosis.

### Time Trends of CAT and Incidence Patterns in Unadjusted Analyses

**Figure 1.**
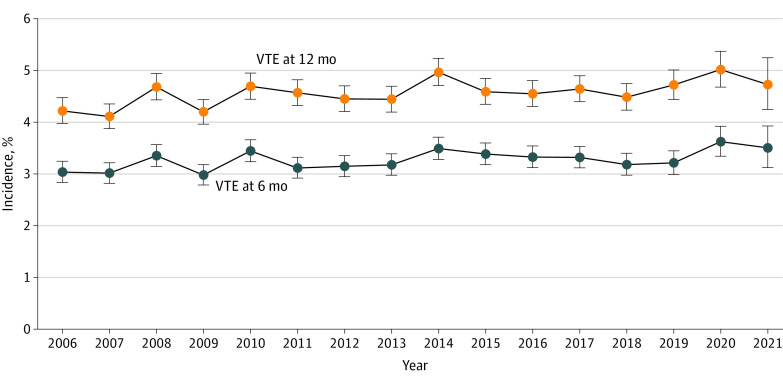
Cumulative Incidence of Venous Thromboembolism (VTE) at 6 and 12 Months After Cancer Diagnosis, 2006-2021 VTE events were captured using a rigorously validated algorithm and based on *International Classification of Diseases, Ninth Revision, Clinical Modification (ICD-9-CM)* and *International Statistical Classification of Diseases, Tenth Revision, Clinical Modification (ICD-10-CM)* codes, anticoagulant medications, and natural language processing radiology reports in inpatient and outpatient encounters. Dots denote means and error bars denote 95% CIs.

**Figure 2.**
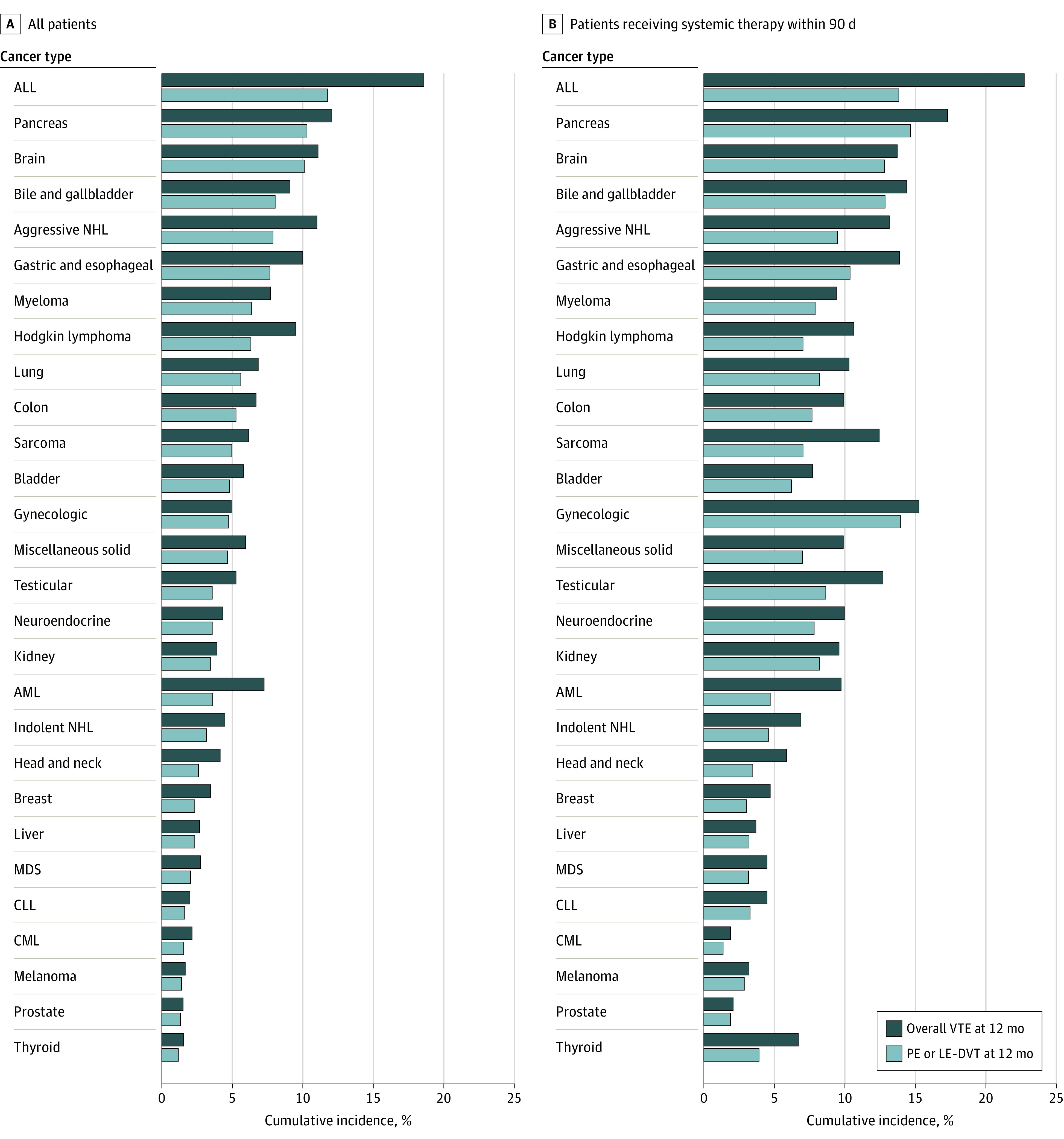
Cumulative Incidence of Venous Thromboembolism (VTE) at 12 Months Stratified by Cancer Type Panel A shows cumulative incidence of VTE at 12 months among all patients in the cohort stratified by cancer type, and panel B shows cumulative incidence of VTE at 12 months among patients who received systemic therapy within 3 months of diagnosis stratified by cancer type. ALL indicates acute lymphoblastic leukemia; AML, acute myeloid leukemia; CLL, chronic lymphocytic leukemia; CML, chronic myeloid leukemia; LE-DVT, lower extremity deep venous thrombosis; MDS, myelodysplastic syndrome; NHL, non-Hodgkin lymphoma; and PE, pulmonary embolism.

Median (IQR) follow-up for CAT assessment was 386 (145-916) days. Cumulative incidence of overall VTE and PE or LE-DVT at 12 months was 4.5% (17 814 patients) and 3.6% (14 237 patients), respectively. Overall, yearly trends of VTE were stable over the 16-year study period, with minor increase from 4.2% (95% CI, 4.0-4.5) in 2006 to 4.7% (95% CI, 4.2-5.2) in 2021 (Figure 1). Cumulative incidence of VTE at 6 and 12 months stratified by diagnosis year can be found in eTable 2 in Supplement 1. As expected, the subset of patients receiving systemic therapy (Figure 2B) had a higher incidence of VTE at 12 months (7.7%) than the overall cohort (4.5%) (Figure 2A). This pattern was particularly pronounced in gynecologic, testicular, and kidney cancers, where the incidence of VTE was 2 to 3 times higher in the treated cohort than the overall cohort (eFigure 2 in Supplement 1).

Among patients with solid tumors, patients with pancreatic cancer (8925 patients [12.1%]), brain cancer (2485 patients [11.1%]), and gastric and esophageal cancer (13 399 patients [10.0%]) had the highest incidence of VTE at 12 months, whereas patients with melanoma (16 702 patients [1.7%]), prostate cancer (133 085 patients [1.5%]), and thyroid cancer (4845 patients [1.6%]) had the lowest incidence of VTE. Among patients with hematologic neoplasms, patients with acute lymphoblastic leukemia (193 patients [18.6%]), aggressive non-Hodgkin lymphomas (5351 patients [11.0%]), and multiple myeloma (6470 patients [7.7%]) had the highest incidence of VTE, whereas patients with myelodysplastic syndrome (4897 patients [2.7%]), chronic myelogenous leukemia (3840 patients [2.1%]), and chronic lymphocytic leukemia (6486 patients [2.0%]) had the lowest incidence of VTE. Although overall VTE and PE or LE-DVT largely correlated among patients with solid tumors, there was significant discrepancy among patients with acute leukemias and aggressive lymphomas, where CR-DVT represented 30% to 50% of all VTE events. Cumulative incidence of VTE stratified by cancer type can be found in eTable 3 in Supplement 1.

### Cancer-Related, Treatment-Related, and Patient-Related Associations With CAT in Adjusted Analyses

In adjusted analysis, cancer type and stage at diagnosis remained the most statistically and clinically significant associations with CAT among patients with solid tumors. The patients at highest risk of VTE were patients with pancreatic cancer (hazard ratio [HR], 6.42; 95% CI, 5.98-6.90), brain tumors (HR, 5.65; 95% CI, 4.96-6.44), and acute lymphoblastic leukemia (HR, 4.98; 95% CI, 3.71-6.68) relative to prostate cancer. Risk of VTE was higher for patients with stage IV cancer (HR, 2.78; 95% CI, 2.68-2.90) compared with patients with stage I cancer, and risk increased progressively by stage (eTable 4 in Supplement 1). Among patients with hematologic neoplasms, we observed higher risk of VTE among patients with aggressive leukemias and lymphomas (HR for aggressive non-Hodgkin lymphoma, 2.65; 95% CI, 2.4-2.89) compared with more indolent diseases such as indolent non-Hodgkin lymphoma (HR, 1.38; 95% CI, 1.26-1.51) (Table 2).

**Table 2.** Multivariable Cox Regression Analysis for Association With Cancer-Associated Thrombosis

Variables	HR (95% CI)	
	Overall VTE incidence	PE or LE-DVT incidence
Age, every 10-y increment	1.02 (1.01-1.04)	1.06 (1.04-1.08)
Sex		
Male	1.15 (1.06-1.24)	1.21 (1.11-1.31)
Female	1 [Reference]	1 [Reference]
Race and ethnicity		
Hispanic	1.04 (0.98-1.10)	0.99 (0.93-1.05)
Non-Hispanic Asian or Pacific Islander	0.84 (0.76-0.93)	0.78 (0.70-0.88)
Non-Hispanic Black	1.23 (1.19-1.27)	1.22 (1.17-1.26)
Non-Hispanic White	1 [Reference]	1 [Reference]
Rurality		
Urban	1 [Reference]	1 [Reference]
Rural	0.90 (0.88-0.93)	0.92 (0.89-0.94)
Unknown	1.05 (0.94-1.18)	1.08 (0.95-1.23)
US region		
Continental	1 [Reference]	1 [Reference]
Southeast	1.00 (0.96-1.05)	1.01 (0.96-1.05)
Midwest	1.05 (1.01-1.09)	1.05 (1.01-1.10)
North Atlantic	0.98 (0.95-1.02)	0.99 (0.94-1.03)
Pacific	1.05 (1.00-1.09)	1.05 (1.00-1.10)
National Area Deprivation Index, percentile		
0-25	1 [Reference]	1 [Reference]
26-50	0.96 (0.92-1.00)	0.96 (0.92-1.01)
51-75	0.95 (0.91-0.99)	0.96 (0.92-1.00)
76-100	0.94 (0.90-0.98)	0.94 (0.90- 0.99)
Unknown	1.05 (0.96-1.14)	1.07 (0.97-1.18)
National Cancer Institute comorbidity index score	0.97 (0.95-1.00)	0.95 (0.92-0.98)
Year of diagnosis	1.01 (1.00-1.01)	1.02 (1.02-1.02)
Khorana score factors		
Body mass index ≥35^a^	1.27 (1.23-1.31)	1.29 (1.25-1.34)
White blood cell count >11 000 cells/μL	1.15 (1.11-1.19)	1.15 (1.10-1.19)
Hemoglobin <10 g/dL	1.14 (1.09-1.19)	1.09 (1.03-1.14)
Platelet count ≥350 × 10^3^/μL	1.13 (1.08-1.17)	1.10 (1.05-1.15)
Additional VTE factors		
History of VTE	2.75 (2.65-2.86)	2.84 (2.72-2.96)
History of paralysis	1.20 (1.08-1.35)	1.13 (0.99-1.29)
Recent hospitalization (90 d)	1.17 (1.13-1.21)	1.18 (1.13-1.22)
Baseline anticoagulant		
None	1 [Reference]	1 [Reference]
Warfarin	1.13 (1.078- 1.19)	1.10 (1.04-1.16)
Low-molecular-weight heparin	1.33 (1.21-1.46)	1.38 (1.25-1.52)
Direct oral anticoagulant	1.02 (0.93-1.12)	1.03 (0.93-1.13)
Baseline antiplatelet		
None	1 [Reference]	1 [Reference]
Aspirin	0.97 (0.94-1.00)	0.95 (0.92-0.99)
P2Y12 inhibitor^b^	1.01 (0.95-1.07)	1.00 (0.94-1.06)
Aspirin and P2Y12 inhibitor^b^	0.98 (0.81-1.18)	0.97 (0.79-1.19)
Other	0.97 (0.84-1.12)	0.96 (0.82-1.13)
Cancer type		
Prostate	1 [Reference]	1 [Reference]
Chronic myeloid leukemia	0.57 (0.49-0.66)	0.51 (0.43-0.60)
Chronic lymphocytic leukemia	0.77 (0.68-0.87)	0.72 (0.63-0.83)
Myelodysplastic syndrome	0.76 (0.66-0.87)	0.64 (0.55-0.75)
Thyroid	1.05 (0.90-1.22)	0.97 (0.82-1.16)
Head and neck	1.32 (1.24-1.41)	1.01 (0.94-1.09)
Indolent non-Hodgkin lymphoma	1.38 (1.26-1.51)	1.18 (1.06-1.30)
Melanoma	1.38 (1.26-1.52)	1.35 (1.22-1.49)
Multiple myeloma	1.72 (1.57-1.87)	1.60 (1.45-1.76)
Breast	1.85 (1.62-2.10)	1.60 (1.37-1.86)
Liver	1.84 (1.70-2.00)	1.92 (1.76-2.09)
Neuroendocrine	1.97 (1.78-2.18)	1.84 (1.64-2.06)
Hodgkin lymphoma	2.00 (1.68-2.38)	1.55 (1.25-1.92)
Acute myeloid leukemia	2.10 (1.82-2.41)	1.35 (1.12-1.62)
Colon	2.45 (2.31-2.59)	2.31 (2.17-2.45)
Kidney	2.17 (2.02-2.33)	2.12 (1.97-2.29)
Testicular	2.49 (1.91-3.25)	2.18 (1.60-2.98)
Aggressive non-Hodgkin lymphoma	2.65 (2.43-2.89)	2.25 (2.04-2.48)
Bladder	2.76 (2.57-2.96)	2.65 (2.46-2.86)
Sarcoma	2.82 (2.46-3.23)	2.56 (2.20-2.98)
Gynecologic	2.93 (2.35-3.61)	3.39 (2.69-4.27)
Miscellaneous solid	3.21 (2.98-3.47)	2.95 (2.71-3.21)
Lung	3.23 (3.08-3.39)	3.10 (2.94-3.27)
Gastric and esophageal	4.03 (3.78-4.30)	3.65 (3.40-3.93)
Bile and gallbladder	4.38 (3.84-4.98)	4.42 (3.85-5.08)
Acute lymphoblastic leukemia	4.98 (3.71-6.68)	3.37 (2.35-4.85)
Brain	5.65 (4.96-6.44)	6.32 (5.51-7.25)
Pancreas	6.42 (5.98-6.90)	6.45 (5.97-6.97)
Cancer stage		
I	1 [Reference]	1 [Reference]
II	1.47 (1.41-1.54)	1.43 (1.37-1.50)
III	1.88 (1.80-1.97)	1.82 (1.73-1.90)
IV	2.78 (2.68-2.90)	2.79 (2.67-2.91)
Unknown	1.44 (1.36-1.52)	1.40 (1.32-1.48)
First-line systemic therapy within 3 mo^c^		
None	1 [Reference]	1 [Reference]
Chemotherapy	1.44 (1.40-1.49)	1.34 (1.30-1.39)
Immune checkpoint inhibitor	1.49 (1.22-1.82)	1.38 (1.11-1.71)
Targeted therapy	1.21 (1.13-1.30)	1.23 (1.14-1.33)
Endocrine therapy	1.20 (1.12-1.28)	1.18 (1.09-1.26)

Abbreviations: HR, hazard ratio; PE, pulmonary embolism; LE-DVT, lower extremity deep venous thrombosis; VTE, venous thromboembolism.

SI conversion factors: To convert hemoglobin to grams per liter, multiply by 10; platelet count to 10^9^/L, multiply by 1; and white blood cell count to cells × 10^9^/L, multiply by 0.001.

^a^ BMI was calculated as weight in kilograms divided by height in meters squared.

^b^ P2Y12 inhibitors refer to clopidogrel, prasugrel, ticagrelor, and ticlopidine.

^c^ First-line systemic therapy was used as a time-varying covariate in the analysis. Chemotherapy included chemotherapy and/or other concurrent therapy; immune checkpoint inhibitor included immune checkpoint inhibitor therapy and/or other concurrent therapy (excluding chemotherapy); targeted therapy included targeted therapy and/or other concurrent therapy (excluding chemotherapy and immune checkpoint inhibitor therapy); endocrine therapy included only endocrine therapy.

Type of systemic treatment was also associated with risk of VTE, though to a lesser extent in the adjusted analysis. Specifically, chemotherapy-based regimens (HR, 1.44; 95% CI, 1.40-1.49) and immunotherapy-based regimens (HR, 1.49; 95% CI, 1.22-1.82) were associated with the highest risk of VTE relative to no treatment. Targeted therapy (HR, 1.21; 95% CI, 1.13-1.30) and endocrine therapy (HR, 1.20; 95% CI, 1.12-1.28) were also associated with higher VTE risk compared with no treatment, although to a lesser degree (Table 2).

Patients with pertinent Khorana score components, including a BMI of 35 or greater (HR, 1.27; 95% CI, 1.23-1.31), white blood cell count greater than 11 000 cells/μL (HR, 1.15; 95% CI, 1.11-1.19), hemoglobin less than 10 g/dL (HR, 1.14; 95% CI, 1.09-1.19), and platelet count greater than or equal to 350 × 10^3^/μL (HR, 1.13; 95% CI, 1.08-1.17) were found to be at statistically higher risk of VTE, although the effect size was modest in the adjusted analysis. Among other recently derived CAT risk model components,^8^ history of VTE was most associated with risk of CAT (HR, 2.75; 95% CI, 2.65-2.86), whereas history of paralysis (HR, 1.20; 95% CI, 1.07-1.35) and recent prolonged hospitalization (HR, 1.17; 95% CI, 1.13-1.21) exhibited modest effect sizes (Table 2).

### Association of Demographics With CAT in Adjusted Analyses

After adjusting for patient-related factors, cancer-related factors, and socioeconomic-related factors, the risk of overall VTE was found to be approximately 20% higher in non-Hispanic Black patients compared with non-Hispanic white patients (HR, 1.23; 95% CI, 1.19-1.27), and approximately 20% lower in Asian or Pacific Islander patients compared with non-Hispanic White patients (HR, 0.84; 95% CI, 0.76-0.93); risk of VTE was similar when comparing Hispanic patients and non-Hispanic White patients (HR, 1.04; 95% CI, 0.98-1.10). Outcomes were consistent when PE or LE-DVT were used as the outcome (Table 2). Cumulative incidence of VTE stratified by race and ethnicity can be found in eTable 5 in Supplement 1. After adjusting for pertinent covariates, non-Hispanic Black patients (HR, 0.92; 95% CI 0.91-0.93), Hispanic patients (HR, 0.81; 95% CI 0.79-0.82), and Asian or Pacific Islander patients (HR, 0.91; 95% CI, 0.88-0.94) had lower risk of death relative to non-Hispanic White patients (eTable 6 in Supplement 1). Despite these generalizations, there was significant heterogeneity by race and ethnicity across cancer types (eFigure 3 in Supplement 1). Other traditional demographic factors, including male sex (HR, 1.15; 95% CI, 1.06-1.24) and age (HR, 1.02; 95% CI, 1.01-1.04]), were also associated with VTE. Interestingly, neighborhood-level socioeconomic factors (national ADI score) and patient comorbidities (NCI-CI) were not associated with CAT but were associated with mortality (eTable 6 in Supplement 1).

## Discussion

We performed a retrospective longitudinal cohort analysis of 434 203 US patients with both solid tumor and hematologic neoplasms over 16 years, investigating the incidence, associated factors, and outcomes of CAT. Using previously validated VTE phenotypes, overall incidence of CAT at 12 months was 4.5% with yearly trends stably ranging between 4.2% to 4.7%. The risk of CAT was most associated with cancer type and stage. The type of systemic treatment was also associated, to a lesser extent, with risk of CAT; higher risk of CAT was observed in patients who received chemotherapy-based and immunotherapy-based regimens. After adjusting for patient-related, cancer-related, and treatment-related variables, we detected a 20% higher risk of CAT among non-Hispanic Black patients and 20% lower risk among Asian or Pacific Islander patients compared with non-Hispanic White patients, although heterogeneities remained between cancer types. These results suggest that patient-specific and treatment-specific factors, accounting for changes in the treatment landscape over time, play a critical role in assessing the risk of CAT, and ongoing efforts to identify these patterns are of utmost importance for risk stratification and prognostic assessment.

In contrast with other large population studies^9,10^ conducted in California and Denmark, the incidence of CAT in our analysis remained largely stable over time. For example, Mulder et al^10^ reported a nearly 3-fold increase in VTE incidence over 20 years, with 12-month VTE incidence ranging from approximately 1% in 1997 to approximately 3.4% in 2017; in contrast, the change in incidence of VTE in our study from 4.2% in 2006 to 4.7% in 2021 appeared less clinically apparent. This discrepancy could be related, in part, to the cancer type compositions in the cohorts, average age, as well as our cohort having predominately male patients. The discrepancy could also be due to reliance on ICD codes to capture VTE events, which over time have evolved. Our rigorously validated outcome algorithm included radiology reports in addition to ICD codes, allowing for more accurate capture and interpretation of CAT trends over time.

Among the factors included in our analysis, we found that cancer type and stage were most significantly associated with CAT, with up to a 6-fold difference seen between cancer subtypes. Although most other studies evaluating trends and associations of risk with CAT have focused primarily on patients with solid tumors, we observed novel patterns among patients with hematologic neoplasms. Specifically, we found a higher incidence of VTE among patients with aggressive vs indolent leukemias and lymphomas. This higher incidence could, in part, be related to cancer subtype and treatment-specific factors. Interestingly, when stratifying further, many of the VTE events in patients with acute myeloid leukemia and acute lymphoblastic leukemia were CR-DVT rather than PE or LE-DVT, perhaps owing to a greater number of peripherally inserted central catheters, which are known to be associated with a higher risk of VTE compared with centrally inserted catheters such as ports.^19^ It is important to note that the pathophysiology of CR-DVT differs from that of PE or LE-DVT. However, the inclusion of CR-DVT events in our analysis allowed for novel insights into factors inherent to those with hematological neoplasms and highlights the need for strategies to mitigate CR-DVT risk.

Systemic therapy was also associated with higher risk of VTE, though to a lesser extent compared with cancer type and stage. Both chemotherapy-based and immunotherapy-based regimens were more associated with CAT compared with targeted agents or endocrine therapy alone. A 2021 study by Moik et al^11^ found high rates of both venous and arterial thrombotic events in patients treated with immune checkpoint inhibitors, with a cumulative incidence rate of 12.9% and 1.8%, respectively, with median follow-up of 8.5 months. Mulder et al^10^ observed an elevated risk of VTE with chemotherapy (subdistribution HR [SHR], 3.35; 95% CI, 3.06-3.66), immunotherapy (SHR, 3.56; 95% CI, 2.75-4.59), and targeted therapy (SHR, 3.85; 95% CI, 3.43-4.32), although there was no substantial risk among patients treated with endocrine therapy alone (SHR, 1.18; 95% CI, 0.99-1.41). It is important to note that unlike our current analysis, these studies^10,11^ often included patients who had received multiple lines of therapy and did not consider time-varying confounding from relapsed or advanced disease.

Notably, when comparing adjusted rates of CAT across patients of different races and ethnicities, we found significantly higher risk of CAT among non-Hispanic Black patients compared with non-Hispanic White patients, despite accounting for socioeconomic and other patient-related and cancer-related factors. Socioeconomic factors such as region, rurality, national ADI score, as well as NCI-CI comorbidity index, were not associated with CAT. Additionally, male sex and age were not found to be associated with risk of CAT, perhaps because our cohort consisted of predominately older male patients. This discrepancy observed among patients of different races and ethnicities is not associated with worse outcomes, because Hispanic patients and Asian or Pacific Islander patients had improved survival outcomes compared with non-Hispanic White patients. These patterns have also been reported in other cancer registry analyses.^13,14,20,21^ For example, an observational analysis^20^ of more than 16 000 patients found an unadjusted incidence rate of CAT to be 3 times higher among non-Hispanic Black patients compared with non-Hispanic White patients. Similarly, another longitudinal cohort analysis^13^ of 15 000 predominantly uninsured patients found a higher risk of CAT among non-Hispanic Black patients (SHR, 1.18; 95% CI, 1.01-1.39) and a lower risk of CAT among Asian or Pacific Islander patients (SHR, 0.58; 95% CI, 0.44-0.77) compared with non-Hispanic White patients, even after adjusting for pertinent factors. Since race and ethnicity are social constructs, these differences in risk of CAT among patients of different races and ethnicities must be interpreted with caution. Observed differences can perhaps be explained by factors including heterogeneity in environmental exposures, differences in the mix of cancer subtypes, and differences in frequency of gene polymorphisms and variants among patients of different races.^22,23,24,25,26^

### Limitations

We acknowledge several limitations of our study. First, we likely underestimated incidence of VTE due to excluding patients with acute VTE within 6 months preceding cancer diagnosis to avoid counting recurrent events, and due to missed events that may have been diagnosed outside the VA system. Despite this limitation, VTE incidence appeared to be higher than in other population-based studies, which was likely due to more sensitive outcome classification. By using rigorously validated ICD and natural language processing algorithms, longitudinal data with limited missing data, and a median follow-up period of more than 1 year, it was possible to accurately interpret VTE trends over time. Our analysis was also limited to patients within 12 months of first cancer diagnosis (consistent with typical practice in studies using data from the Surveillance, Epidemiology, and End Results Program), and we were not able to include Eastern Cooperative Oncology Group performance status or the presence of central venous catheters as potential factors associated with the risk of CAT. Furthermore, we acknowledge that our study population predominantly consisted of an older, male population with robust access to health care through their veteran benefits, which may not fully reflect associations of risk among all patients with cancer. We also acknowledge that interpretation of baseline anticoagulant use as a variable is likely confounded by those receiving treatment for subacute VTE or other comorbidities such as atrial fibrillation, rather than a population of patients receiving primary prophylaxis. Additionally, we chose to include patients who received upfront systemic therapy (for neoadjuvant, adjuvant, or metastatic disease) within the first 3 months of treatment to avoid mixing the effect of subsequent line therapy. However, among patients receiving systemic therapy, the majority received it within 3 months.

## Conclusions

In this cohort study of patients with both solid tumor and hematologic neoplasms, we observed a high incidence of CAT with yearly trends that remained relatively stable over the 16-year study period. As with other studies, cancer type, staging, and systemic treatment regimen were significantly associated with VTE risk. Patients with aggressive hematologic neoplasms had a higher incidence of VTE compared with patients with indolent neoplasms, although this trend may be associated in part with catheter-related events. Patients of different races and ethnicities continued to demonstrate discrepant risk of VTE, whereas socioeconomic and other demographic factors had minimal impact. Taken together, these findings highlight several additional epidemiologic factors that warrant consideration for future VTE risk stratification models.

## Notes

Supplement 1. **eMethods.** Data Extraction and Harmonization for the Veterans Affairs Healthcare System
eReferences.
**eTable 1.** Systemic Therapy Classifications **eTable 2.** Cumulative Incidence of VTE at 6 and 12 Months by Diagnosis Year **eTable 3.** Cumulative Incidence of VTE at 1 Year by Cancer Type **eTable 4.** Cumulative Incidence of VTE at 1 Year by Cancer Stage **eTable 5.** Cumulative Incidence of VTE at 1 Year by Race and Ethnicity **eTable 6.** Multivariable Cox Regression Analysis for Association With Overall Mortality **eFigure 1.** Consort Diagram **eFigure 2.** Cumulative Incidence of VTE at 1 Year by Cancer Stage **eFigure 3.** Cumulative Incidence of VTE At 1 Year by Cancer Type and Race and Ethnicity

Supplement 2.
Data Sharing Statement
