# Supplementary material for: Epidemiology of Cancer-Associated Venous Thromboembolism in Patients With Solid and Hematologic Neoplasms in the Veterans Affairs Health Care System
Source: JAMA Netw Open. 2023 Jun 12;6(6):e2317945. doi: 10.1001/jamanetworkopen.2023.17945 (PMC10261992; doi:10.1001/jamanetworkopen.2023.17945)
Supplement: Supplement 1. — eMethods. Data Extraction and Harmonization for the Veterans Affairs Healthcare System eReferences. eTable 1. Systemic Therapy Classifications eTable 2. Cumulative Incidence of VTE at 6 and 12 Months by Diagnosis Year eTable 3. Cumulative Incidence of VTE at 1 Year by Cancer Type eTable 4. Cumulative Incidence of VTE at 1 Year by Cancer Stage eTable 5. Cumulative Incidence of VTE at 1 Year by Race and Ethnicity eTable 6. Multivariable Cox Regression Analysis for Association With Overall Mortality eFigure 1. Consort Diagram eFigure 2. Cumulative Incidence of VTE at 1 Year by Cancer Stage eFigure 3. Cumulative Incidence of VTE At 1 Year by Cancer Type and Race and Ethnicity [file jamanetwopen-e2317945-s001.pdf]

Supplemental Online Content

Martens KL, Li A, La J, et al. Epidemiology of cancer-associated venous thromboembolism in patients with solid and hematologic neoplasms in the Veterans Affairs Health Care System. *JAMA Netw Open*. 2023;6(6):e2317945. doi:10.1001/jamanetworkopen.2023.17945

eMethods. Data Extraction and Harmonization for the Veterans Affairs Healthcare System
eReferences.
eTable 1. Systemic Therapy Classifications
eTable 2. Cumulative Incidence of VTE at 6 and 12 Months by Diagnosis Year
eTable 3. Cumulative Incidence of VTE at 1 Year by Cancer Type
eTable 4. Cumulative Incidence of VTE at 1 Year by Cancer Stage
eTable 5. Cumulative Incidence of VTE at 1 Year by Race and Ethnicity
eTable 6. Multivariable Cox Regression Analysis for Association With Overall Mortality
eFigure 1. Consort Diagram
eFigure 2. Cumulative Incidence of VTE at 1 Year by Cancer Stage
eFigure 3. Cumulative Incidence of VTE At 1 Year by Cancer Type and Race and Ethnicity

This supplemental material has been provided by the authors to give readers additional information about their work.

© 2023 Martens KL et al. *JAMA Network Open.*

eMethods. Data extraction and harmonization for the Veterans Affairs healthcare system

Data source and linkage

This study utilizes patient-level data from the Veterans Affairs (VA) healthcare system, which is the largest integrated healthcare system with 171 medical centers and 1,113 outpatient clinics for veterans in the United States (US).

Linkage at the patient level was achieved by matching unique identifiers between electronic health record data collated in the VA Corporate Data Warehouse (CDW) and national VA Cancer Registry data, which is also available in the CDW (oncology domain) from 2006-2021.

Data extracted from the VA Cancer Registry included cancer histology, American Joint Committee on Cancer (AJCC) staging, demographics, and mortality. Data extracted from the CDW included socioeconomic factors, clinical encounter, medical history, problem list, medication, International Classification of Diseases Clinical Modification (ICD-9-CM and ICD-10-CM) diagnosis and procedure codes, Current Procedural Terminology/Healthcare Common Procedure Coding System (CPT/HCPCS) procedure codes, discharge summary, clinic notes, and radiology reports.

Cohort selection (eFigure 1)

Cancer Registry exclusions required patients to have a first-ever histologically confirmed invasive cancer diagnosis. CDW exclusions required patients to be primary user, and not having had recent diagnosis of acute venous thromboembolism (VTE) in the preceding 180 days (eFigure 1).

Index date and follow-up

Outcome was evaluated from the index date of histologic cancer diagnosis until first outcome event, death, loss of follow-up defined as a 90-day gap without any clinical encounters, or administrative censoring on 4/1/2022.

Outcome variables

<u>Primary outcome</u>: *overall VTE* was defined as a composite outcome of radiologically confirmed symptomatic or incidental pulmonary embolism (PE), proximal or distal lower extremity DVT (LE-DVT), or upper extremity catheter-related DVT (CR-DVT) after index date.

<u>Secondary outcome</u>: *PE/LE-DVT w*as defined as an important subgroup of the primary outcome (excluding CR-DVT). Please refer to the online Data Supplement from our previous publication for detailed cohort definition and validation where the weighted sensitivity was 96% and weighted positive predicted value was 91% [1].

Baseline variables

<u>Cancer site/histology subtype:</u>

Cancer site and histology were recoded from VA Cancer Registry at the time of diagnosis according to the SEER standard ICD-O-3 recode for common solid tumors and rare disease classification for hematologic malignancies and rare histology like leukemia, lymphoma, sarcoma and neuroendocrine [2, 3].

<u>Cancer Stage:</u>

AJCC cancer staging (I, II, III, IV, unknown) at the time of cancer diagnosis was extracted from the VA Cancer Registry. Certain cancers had unknown stage when AJCC classification was not appropriate (e.g. brain cancer, leukemia, myeloma) for which SEER staging was used.

<u>Treatment Regimen (supplemental Table 2):</u>

First-line systemic therapy extracted from the VA CDW and classified as chemotherapy, immune checkpoint inhibitor, target therapy, or endocrine therapy. See supplemental Table 2 for detailed breakdown by generic drug name.

<u>Age:</u>

Age in years was extracted directly from the CDW at the time of cancer diagnosis.

<u>Sex:</u>

Sex was extracted directly from the CDW at the time of cancer diagnosis.

© 2023 Martens KL et al. *JAMA Network Open.*

Race/Ethnicity:
Race/ethnicity was extracted from the VA Cancer Registry and CDW (SPatient) and recoded as Non-Hispanic White, Non-Hispanic Black, Hispanic, Non-Hispanic Asian, Pacific Islander, Alaskan Natives, American Indian (altogether classified as API), or Other/Unknown.

Region and Rurality:
Region and rurality were extracted directly from the CDW at the time of cancer diagnosis.

National Area Deprivation Index (ADI) score:
To determine socioeconomic status, patients' home addresses were geocoded and linked to corresponding census block groups. These block groups were mapped to the area deprivation index (ADI), which is derived using 17 weighted indicators from US Census American Community Survey 5-year estimates (poverty, housing, employment, education) and divided into quartiles based on the national ADI scores distribution, with higher scores indicating more disadvantaged neighborhoods [4, 5].

National Cancer Institute Comorbidity Index (NCI-CI):
Individual comorbidities, including history of paralysis, were determined from the NCI-CI within a 12-month lookback window before the diagnosis date using inpatient and outpatient facility coded diagnoses extracted from the VA CDW [6].

History of PE/LE-DVT:
ICD codes for VTE any time prior to diagnosis were extracted from the VA CDW. These were not confirmed with chart review. Please refer to the online Data Supplement from our previous publication for exact codes and timing use [1].

Prolonged hospitalization:
Prolonged hospitalization ≥3 days was extracted from the VA CDW prior to diagnosis within a 3-month lookback window.

Anticoagulation/antiplatelet prescription:
Anticoagulation/antiplatelet prescription data was extracted from the VA CDW prior to diagnosis within a 6-month lookback window from the outpatient settings.

Body Mass Index:
Data calculated from the VA CDW using any height and the closest weight prior to diagnosis within a 36-month lookback window.

White blood cell (WBC) count:
Data captured from the VA CDW using the closest WBC within a window between 3-month prior to 1-month after the diagnosis date. If multiple values were present, then the one closest to the index date was used.

Hemoglobin:
Data captured from the VA CDW using the closest hemoglobin within a window between 3-month prior to 1-month after the diagnosis date. If multiple values were present, then the one closest to the index date was used.

Platelet count:
Data captured from the VA CDW using the closest platelet count within a window between 3-month prior to 1-month after the diagnosis date. If multiple values were present, then the one closest to the index date was used.

Missing data
Missing data were classified as a categorical variable "NA" in our study, so the final multivariable model is run on the entire dataset without imputation or exclusion.

© 2023 Martens KL et al. *JAMA Network Open.*

eReferences

1. Li A, La J, May SB, et al. Derivation and Validation of a Clinical Risk Assessment Model for Cancer-Associated Thrombosis in Two Unique US Health Care Systems. J Clin Oncol. 2023 Jan 10:JCO2201542.
2. National Cancer Institute (NCI): Site Recode ICD-O-3/WHO 2008 Definition [Internet]. Survuillance, Epidemiol End Results Progr , 2021[cited 2023 Feb 1] Available from:https://seer.cancer.gov/siterecode/icdo3_dwhoheme/index.html
3. National Cancer Institute (NCI): Rare Cancer Classification [Internet]. Survuillance, Epidemiol End Results Progr , 2021[cited 2023 Feb 1] Available from: https://seer.cancer.gov/seerstat/variables/seer/raresiterecode/
4. Singh, G.K. Area deprivation and widening inequalities in US mortality, 1969-1998. Am J Public Health, 2003. 93(7): p. 1137-43.
5. Kind, A.J.H. and W.R. Buckingham. *Making Neighborhood-Disadvantage Metrics Accessible - The Neighborhood Atlas*. N Engl J Med 2018. 378(26): p. 2456-2458.
6. National Cancer Institute (NCI): NCI Comorbidity Index 2021 Version [Internet]. Div Cancer Control Popul Sci 2021; Available from: https://healthcaredelivery.cancer.gov/seermedicare/considerations/macro-2021.html.

© 2023 Martens KL et al. *JAMA Network Open.*

eTable 1. Systemic Therapy Classifications

Drug name (generic)	Drug class (proposed)
abemaciclib	target
abiraterone	endo
acalabrutinib	target
ado-trastuzumab	target
afatinib	target
alectinib	target
alitretinoin	target
alpelisib	target
anastrozole	endo
arsenic	target
asparaginase	chemo
atezolizumab	immuno
avelumab	immuno
axitinib	target
azacitidine	chemo
bendamustine	chemo
bevacizumab	target
bexarotene	target
bicalutamide	endo
binimetinib	target
bleomycin	chemo
blinatumomab	target
bortezomib	target
bosutinib	target
brentuximab	target
cabazitaxel	chemo
cabozantinib	target
capecitabine	chemo
carboplatin	chemo
carfilzomib	target
carmustine	chemo
cemiplimab-rwlc	immuno
ceritinib	target
cetuximab	target
chlorambucil	chemo
cisplatin	chemo
cladribine	chemo
clofarabine	chemo
cobimetinib	target
crizotinib	target
cyclophosphamide	chemo
cytarabine	chemo
cytarabine/PF	chemo
dabrafenib	target
dacarbazine	chemo
dactinomycin	chemo
daratumumab	target
darolutamide	endo
dasatinib	target
daunorubicin	chemo
decitabine	chemo

© 2023 Martens KL et al. *JAMA Network Open.*

degarelix	endo
docetaxel	chemo
doxorubicin	chemo
durvalumab	immuno
elotuzumab	target
enasidenib	target
encorafenib	target
enzalutamide	endo
epirubicin	chemo
erdafitinib	target
eribulin	chemo
erlotinib	target
etoposide	chemo
everolimus	target
exemestane	endo
fam-trastuzumab	target
fludarabine	chemo
fluorouracil	chemo
flutamide	endo
fulvestrant	endo
gemcitabine	chemo
gemtuzumab	target
goserelin	endo
hydroxyurea	chemo
ibrutinib	target
idarubicin	chemo
idelalisib	target
ifosfamide	chemo
imatinib	target
inotuzumab	target
interferon	target
irinotecan	chemo
ivosidenib	target
ixabepilone	chemo
ixazomib	target
lapatinib	target
larotrectinib	target
lenalidomide	target
lenvatinib	target
letrozole	endo
leuprolide	endo
lomustine	chemo
lorlatinib	target
mercaptopurine	chemo
methotrexate	chemo
midostaurin	target
mitomycin	chemo
mitotane	endo
mitoxantrone	chemo
nelarabine	chemo
neratinib	target
nilotinib	target
niraparib	target

© 2023 Martens KL et al. *JAMA Network Open.*

nivolumab	immuno
obinutuzumab	target
ofatumumab	target
olaparib	target
olaratumab	target
osimertinib	target
oxaliplatin	chemo
paclitaxel	chemo
palbociclib	target
panitumumab	target
panobinostat	target
pazopanib	target
pegaspargase	chemo
peginterferon	target
pembrolizumab	immuno
pemetrexed	chemo
pertuzumab	target
polatuzumab	target
pomalidomide	target
ponatinib	target
procarbazine	chemo
ramucirumab	target
regorafenib	target
ribociclib	target
rituximab	target
romidepsin	target
ruxolitinib	target
sacituzumab	target
selpercatinib	target
sorafenib	target
sunitinib	target
tafasitamab-cxix	target
talazoparib	target
tamoxifen	endo
temozolomide	chemo
temsirolimus	target
thiotepa	chemo
topotecan	chemo
trabectedin	chemo
trametinib	target
trastuzumab	target
trastuzumab-anns	target
tretinoin	target
trifluridine/tipiracil	chemo
triptorelin	endo
tucatinib	target
vemurafenib	target
venetoclax	target
vinblastine	chemo
vincristine	chemo
vinorelbine	chemo
vismodegib	target
vorinostat	target

© 2023 Martens KL et al. *JAMA Network Open.*

alemtuzumab	target
capmatinib	target
daratumumab-hyaluronidase-fihj	target
decitabine/cedazuridine	target
enfortumab	target
gilteritinib	target
ipilimumab	immuno
lurbinectedin	chemo
mechlorethamine	chemo
melphalan	chemo
pemigatinib	target
pentostatin	chemo
pertuzumab-trastuzumab-hy-zzxf	target
ripretinib	target
rucaparib	target
siltuximab	target
sonidegib	target
valrubicin	chemo
vandetanib	target
gefitinib	target
nilutamide	endo
thioguanine	chemo
toremifene	endo

© 2023 Martens KL et al. *JAMA Network Open.*

eTable 2. Cumulative Incidence of VTE at 6 and 12 Months by Diagnosis Year

Diagnosis year	N (%)	6-month CI VTE	12-month CI VTE
2006	28,121 (6.5%)	3.0%	4.2%
2007	30,174 (6.9%)	3.0%	4.1%
2008	29,954 (6.9%)	3.4%	4.7%
2009	30,405 (7.0%)	3.0%	4.2%
2010	30,050 (6.9%)	3.4%	4.7%
2011	30,393 (7.0%)	3.1%	4.6%
2012	29,696 (6.8%)	3.1%	4.5%
2013	29,068 (6.7%)	3.2%	4.4%
2014	29,165 (6.7%)	3.5%	5.0%
2015	29,939 (6.9%)	3.4%	4.6%
2016	29,654 (6.8%)	3.3%	4.6%
2017	30,325 (7.0%)	3.3%	4.6%
2018	27,931 (6.4%)	3.2%	4.5%
2019	24,057 (5.5%)	3.2%	4.7%
2020	16,850 (3.9%)	3.6%	5.0%
2021	8,421 (1.9%)	3.5%	4.7%

Abbreviations: VTE, venous thromboembolism; CI, cumulative incidence

© 2023 Martens KL et al. *JAMA Network Open.*

eTable 3. Cumulative Incidence of VTE at 1 Year by Cancer Type
(A) Among all patients (n= 434,203)

**Cancer Type**	**N**	**CI VTE**		**CI PE/LE-DVT**	
		**6 months**	**12 months**	**6 months**	**12 months**
ALL	193	16.2%	18.6%	10.0%	11.8%
pancreas	8,925	9.8%	12.1%	8.4%	10.3%
brain	2,485	9.0%	11.1%	8.2%	10.1%
bile & gallbladder	2,546	6.7%	9.1%	5.7%	8.1%
aggressive NHL	5,351	8.9%	11.0%	6.3%	7.9%
upper GI	13,399	7.6%	10.0%	5.8%	7.7%
myeloma	6,470	4.9%	7.7%	3.9%	6.4%
Hodgkin lymphoma	1,129	6.7%	9.5%	4.3%	6.3%
lung	7,5441	5.2%	6.9%	4.2%	5.6%
lower GI	29,580	4.7%	6.7%	3.7%	5.3%
sarcoma	3,017	4.4%	6.2%	3.4%	5.0%
bladder	15,040	3.8%	5.8%	3.1%	4.8%
gynecologic	1,246	3.1%	4.9%	3.0%	4.7%
miscellaneous solid	13,763	4.7%	6.0%	3.7%	4.7%
testicular	1,006	4.0%	5.3%	3.0%	3.6%
neuroendocrine	6,831	3.2%	4.3%	2.7%	3.6%
kidney	18,315	3.0%	3.9%	2.6%	3.5%
AML	2,657	6.4%	7.3%	3.1%	3.6%
indolent NHL	8,987	3.3%	4.5%	2.3%	3.2%
head & neck	25,486	3.0%	4.1%	2.0%	2.6%
breast	5,075	1.9%	3.4%	1.2%	2.4%
liver	17,406	1.8%	2.7%	1.6%	2.3%
MDS	4,897	1.6%	2.7%	1.1%	2.0%
CLL	6,486	1.1%	2.0%	0.9%	1.6%
CML	3,840	1.0%	2.1%	0.8%	1.6%
melanoma	16,702	0.9%	1.7%	0.8%	1.4%
prostate	133,085	0.9%	1.5%	0.8%	1.3%
thyroid	4,845	1.0%	1.6%	0.8%	1.2%

© 2023 Martens KL et al. *JAMA Network Open.*

(B) Among patients treated with systemic therapy within first 90 days (n= 118,731)

Cancer Type	N	CI VTE		CI PE/LE-DVT	
		6 months	12 months	6 months	12 months
ALL	147	20.5%	22.7%	12.3%	13.8%
pancreas	3611	13.4%	17.3%	11.3%	14.7%
brain	1351	10.9%	13.7%	10.3%	12.8%
bile & gallbladder	696	10.6%	14.4%	9.1%	12.8%
aggressive NHL	3819	10.9%	13.2%	7.7%	9.5%
upper GI	6190	10.5%	13.9%	7.7%	10.4%
myeloma	3594	5.9%	9.4%	4.9%	7.9%
Hodgkin lymphoma	788	8.1%	10.6%	5.1%	7.0%
lung	24938	7.9%	10.3%	6.2%	8.2%
lower GI	8737	6.8%	9.9%	5.2%	7.7%
sarcoma	320	11.1%	12.5%	6.3%	7.0%
bladder	4667	5.4%	7.7%	4.3%	6.2%
gynecologic	154	12.5%	15.3%	11.1%	13.9%
miscellaneous solid	3598	7.8%	9.9%	5.4%	7.0%
testicular	264	11.3%	12.7%	8.1%	8.6%
neuroendocrine	1216	8.0%	10.0%	6.2%	7.8%
kidney	1541	7.8%	9.6%	6.8%	8.2%
AML	1564	8.8%	9.7%	4.1%	4.7%
indolent NHL	3609	5.4%	6.9%	3.5%	4.6%
head & neck	9111	4.5%	5.9%	2.7%	3.5%
breast	1971	3.1%	4.7%	1.9%	3.0%
liver	4839	2.5%	3.7%	2.2%	3.2%
MDS	1227	2.9%	4.5%	1.9%	3.2%
CLL	769	3.6%	4.5%	2.5%	3.3%
CML	2635	0.9%	1.9%	0.7%	1.4%
melanoma	1332	2.3%	3.2%	1.9%	2.9%
prostate	25931	1.1%	2.1%	1.0%	1.9%
thyroid	112	3.6%	6.7%	1.8%	3.9%

Abbreviations: CI, cumulative incidence; VTE, venous thromboembolism; PE/LE-DVT, pulmonary embolism / lower-extremity deep venous thrombosis; ALL, acute lymphocytic leukemia; NHL, non-Hodgkin lymphoma; GI, gastrointestinal; AML, acute myeloid leukemia; MDS, myelodysplastic syndrome; CLL, chronic lymphocytic leukemia; CML, chronic myeloid leukemia

© 2023 Martens KL et al. *JAMA Network Open.*

eTable 4. Cumulative Incidence of VTE by Cancer Stage

Cancer Type	Stage I-II	Stage III-IV
ALL	29 (13.8%)	164 (19.3%)
pancreas	3123 (9.9%)	5802 (13.2%)
brain	2437 (11.0%)	48 (14.7%)
bile & gallbladder	1362 (7.7%)	1184 (10.8%)
aggressive NHL	2137 (9.4%)	3214 (12.1%)
upper GI	5652 (8.4%)	7747 (11.2%)
myeloma	0 (0.0%)	6470 (7.7%)
Hodgkin lymphoma	524 (10.2%)	605 (8.9%)
lung	29152 (4.6%)	46289 (8.2%)
lower GI	17279 (4.8%)	12301 (9.0%)
sarcoma	2303 (5.0%)	714 (9.7%)
bladder	12411 (4.0%)	2629 (13.6%)
gynecologic	951 (2.6%)	295 (12.1%)
miscellaneous solid	11685 (5.7%)	2078 (7.5%)
testicular	918 (3.6%)	88 (22.0%)
neuroendocrine	4579 (3.4%)	2252 (6.2%)
kidney	13747 (2.6%)	4568 (7.9%)
AML	0 (0.0%)	2657 (7.3%)
indolent NHL	4052 (3.1%)	4935 (5.6%)
head & neck	10655 (2.5%)	14831 (5.2%)
breast	4309 (2.9%)	766 (6.3%)
liver	12158 (2.1%)	5248 (4.0%)
MDS	0 (0.0%)	4897 (2.7%)
CLL	951 (1.4%)	5535 (2.1%)
CML	0 (0.0%)	3840 (2.1%)
melanoma	14825 (1.2%)	1877 (4.8%)
prostate	112538 (1.3%)	20547 (2.8%)
thyroid	3760 (1.2%)	1085 (2.9%)

Abbreviations: VTE, venous thromboembolism; ALL, acute lymphocytic leukemia; NHL, non-Hodgkin lymphoma; GI, gastrointestinal; AML, acute myeloid leukemia; MDS, myelodysplastic syndrome; CLL, chronic lymphocytic leukemia; CML, chronic myeloid leukemia

eTable 5. Cumulative Incidence of VTE by Race and Ethnicity[a]

Cancer Type	Hispanic	Non-Hispanic API	Non-Hispanic Black	Non-Hispanic White
ALL	15 (0.0%)	0 (0.0%)	17 (0.0%)	154 (17.5%)
pancreas	417 (11.1%)	166 (10.1%)	1,720 (16.2%)	6,508 (11.2%)
brain	119 (13.0%)	42 (0.0%)	274 (14.4%)	2,019 (10.7%)
bile & gallbladder	195 (8.9%)	47 (0.0%)	455 (11.6%)	1,819 (8.7%)
aggressive NHL	343 (9.7%)	127 (13.7%)	715 (12.8%)	4,113 (10.8%)
upper GI	700 (10.0%)	241 (7.5%)	2,238 (11.0%)	10,074 (9.8%)
myeloma	344 (7.3%)	132 (4.2%)	2,014 (7.9%)	3,903 (7.8%)
Hodgkin lymphoma	77 (0.0%)	23 (0.0%)	208 (6.7%)	809 (11.1%)
lung	1,704 (9.0%)	1,112 (5.8%)	12,054 (9.0%)	59,860 (6.4%)
lower GI	1,791 (7.1%)	611 (5.3%)	5,388 (8.4%)	21,505 (6.2%)
sarcoma	175 (6.9%)	59 (0.0%)	516 (9.0%)	2,241 (5.6%)
bladder	517 (5.3%)	190 (6.1%)	1,416 (7.5%)	12,779 (5.6%)
gynecologic	56 (0.0%)	39 (0.0%)	248 (3.1%)	888 (5.9%)
miscellaneous solid	664 (6.7%)	228 (3.7%)	2,226 (7.3%)	10,516 (5.7%)
testicular	105 (4.5%)	22 (0.0%)	64 (0.0%)	807 (5.6%)
neuroendocrine	355 (5.3%)	113 (5.3%)	1,519 (4.2%)	4,779 (4.3%)
kidney	1,157 (3.6%)	382 (5.2%)	3,700 (3.2%)	12,898 (4.1%)
AML	114 (10.7%)	53 (0.0%)	334 (7.2%)	2,130 (6.9%)
indolent NHL	467 (5.2%)	152 (4.1%)	1,134 (4.8%)	7,152 (4.4%)
head & neck	962 (5.0%)	377 (5.1%)	3,885 (4.8%)	20,006 (4.0%)
breast	241 (2.3%)	145 (0.8%)	1,411 (3.9%)	3,225 (3.4%)
liver	1,588 (3.1%)	381 (1.7%)	4,281 (2.5%)	10,984 (2.7%)
MDS	206 (3.1%)	75 (0.0%)	532 (3.5%)	4,041 (2.6%)
CLL	197 (2.4%)	88 (0.0%)	743 (2.4%)	5,396 (2.0%)
CML	177 (0.6%)	68 (0.0%)	614 (2.0%)	2,944 (2.3%)
melanoma	194 (3.7%)	168 (1.7%)	119 (4.8%)	16,094 (1.6%)
prostate	6,952 (1.1%)	2,221 (0.9%)	40,853 (1.6%)	81,932 (1.5%)
thyroid	361 (1.3%)	152 (2.3%)	693 (1.6%)	3,581 (1.5%)

[a]Race/ethnicity groups with fewer than 100 events by specific cancer type were excluded from representation.
Abbreviations: VTE, venous thromboembolism; API, Asian Pacific Islander; ALL, acute lymphocytic leukemia; NHL, non-Hodgkin lymphoma; GI, gastrointestinal; AML, acute myeloid leukemia; MDS, myelodysplastic syndrome; CLL, chronic lymphocytic leukemia; CML, chronic myeloid leukemia

© 2023 Martens KL et al. *JAMA Network Open.*

eTable 6. Multivariable Cox Regression Analysis for Association With Overall Mortality

**Variables**	**Overall VTE HR (95% CI)**
**Age**, every 10-year increase	1.47 (1.47 - 1.48)
**Male sex**	1.24 (1.21 - 1.28)
**Race/Ethnicity**	
Non-Hispanic White	Ref.
Non-Hispanic Black	0.92 (0.91 - 0.93)
Hispanic	0.81 (0.79 - 0.82)
Non-Hispanic API	0.91 (0.88 - 0.94)
**Rurality**	
Urban	Ref.
Rural	0.97 (0.96 - 0.98)
Unknown	1.04 (1.00 - 1.08)
**Region**	
Continental	Ref.
Southeast	0.97 (0.95 - 0.98)
Midwest	0.96 (0.95 - 0.98)
North Atlantic	0.93 (0.92 - 0.95)
Pacific	0.99 (0.98 - 1.01)
**National ADI**	
0-25%	Ref.
26-50%	1.03 (1.01 - 1.04)
51-75%	1.06 (1.04 - 1.07)
76-100%	1.15 (1.13 - 1.16)
Unknown	1.11 (1.07 - 1.14)
**NCI-CI**	1.50 (1.49 - 1.51)
**Year of diagnosis**	0.98 (0.98 - 0.98)
**Khorana Score Predictors**	
BMI $\geq$ 35 kg/m^2	0.93 (0.92 - 0.94)
WBC > 11 x 10^9/L	1.28 (1.27 - 1.30)
Hgb < 10 g/dL	1.37 (1.35 - 1.39)
Platelet $\geq$ 350 × 10^9/L	1.29 (1.27 - 1.31)
**Additional VTE predictors**	
History of VTE	1.11 (1.09 - 1.13)
History of paralysis	1.09 (1.05 - 1.14)
Recent hospitalization (90d)	1.45 (1.43 - 1.46)
**Baseline Anticoagulant**	
None	Ref.
Warfarin	1.07 (1.05 - 1.09)
LMWH	0.96 (0.92 - 1.00)
DOAC	0.96 (0.93 - 1.00)
**Baseline Antiplatelet**	
None	Ref.
Aspirin	1.05 (1.04 - 1.06)
P2Y12 inhibitor	1.03 (1.01 - 1.05)
Aspirin + P2Y12 inhibitor	0.97 (0.91 - 1.03)
Other	1.09 (1.04 - 1.14)
**Cancer Site**	
Prostate	Ref.
CML	0.50 (0.48 - 0.53)
CLL	0.50 (0.48 - 0.52)
Thyroid	0.87 (0.82 - 0.92)
Indolent NHL	1.01 (0.97 - 1.04)

© 2023 Martens KL et al. *JAMA Network Open.*

Multiple myeloma	1.01 (0.98 - 1.05)
Hodgkin lymphoma	1.06 (0.97 - 1.16)
MDS	1.11 (1.07 - 1.15)
Testicular	1.24 (1.05 - 1.45)
Aggressive NHL	1.51 (1.45 - 1.56)
Melanoma	1.67 (1.63 - 1.72)
Breast	1.72 (1.63 - 1.82)
Head & neck	1.95 (1.91 - 2.00)
Kidney	1.97 (1.92 - 2.02)
Lower GI	2.00 (1.96 - 2.04)
Neuroendocrine	2.08 (2.00 - 2.15)
Gynecologic	2.27 (2.05 - 2.51)
Sarcoma	2.45 (2.33 - 2.57)
ALL	2.54 (2.17 - 2.96)
AML	2.84 (2.72 - 2.97)
Bladder	2.86 (2.79 - 2.93)
Miscellaneous solid	3.97 (3.87 - 4.07)
Lung	5.08 (5.00 - 5.16)
Upper GI	5.52 (5.40 - 5.65)
Bile & gallbladder	6.32 (6.05 - 6.61)
Liver	7.08 (6.92 - 7.23)
Brain	8.09 (7.73 - 8.48)
Pancreas	10.05 (9.79 - 10.31)
**Cancer Stage**	
Stage I	Ref.
Stage II	1.58 (1.56 - 1.61)
Stage III	2.18 (2.15 - 2.21)
Stage IV	5.03 (4.97 - 5.10)
Unknown	2.15 (2.11 - 2.18)
**First-line Systemic Therapy within 3 months**[a]	
None	Ref.
Chemotherapy+	0.89 (0.88 - 0.90)
Immune checkpoint inhibitor+	0.80 (0.74 - 0.86)
Targeted therapy+	0.94 (0.92 - 0.96)
Endocrine therapy	1.32 (1.29 - 1.35)

Abbreviations: VTE, venous thromboembolism; HR, hazard ratio; CI, confidence interval; API, Asian Pacific Islander; ADI, area deprivation index; BMI, body mass index; WBC, white blood cell; Hgb, hemoglobin; NCI-CI, National Cancer Institute comorbidity index; LMWH, low molecular weight heparin; DOAC, direct oral anticoagulant; CML, chronic myeloid leukemia; CLL, chronic lymphocytic leukemia; NHL, non-Hodgkin lymphoma; MDS, myelodysplastic syndrome; GI, gastrointestinal; ALL, acute lymphocytic leukemia; AML, acute myeloid leukemia

[a]First-line systemic therapy was treated as a time-varying covariate in the analysis

© 2023 Martens KL et al. *JAMA Network Open.*

eFigure 1. Consort Diagram

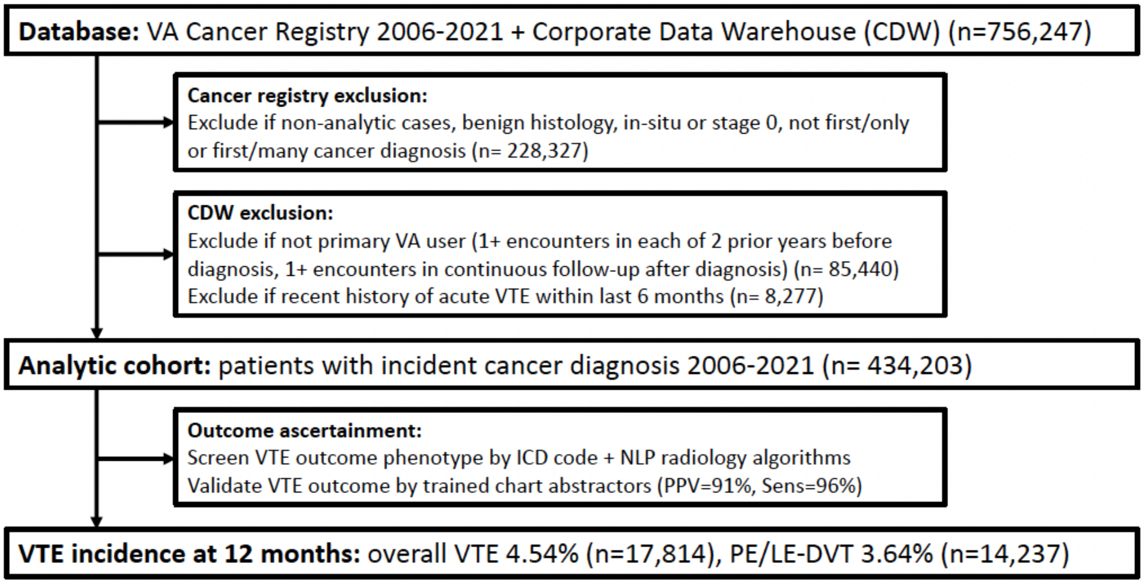

Patient linkage, data harmonization, cohort exclusion, and variable extraction are described in detail in data supplemental Method 1.

© 2023 Martens KL et al. *JAMA Network Open.*

eFigure 2. Cumulative Incidence of VTE at 1 Year by Cancer Stage

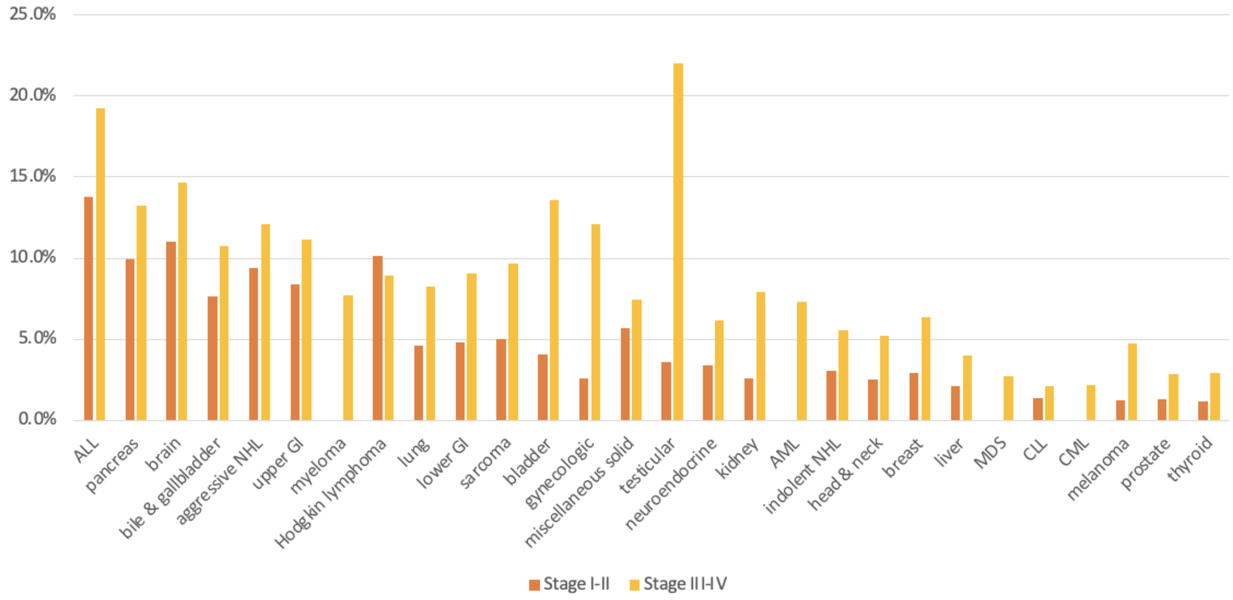

© 2023 Martens KL et al. *JAMA Network Open.*

eFigure 3: Cumulative incidence of VTE at 1-year by cancer type and race/ethnicity[a]

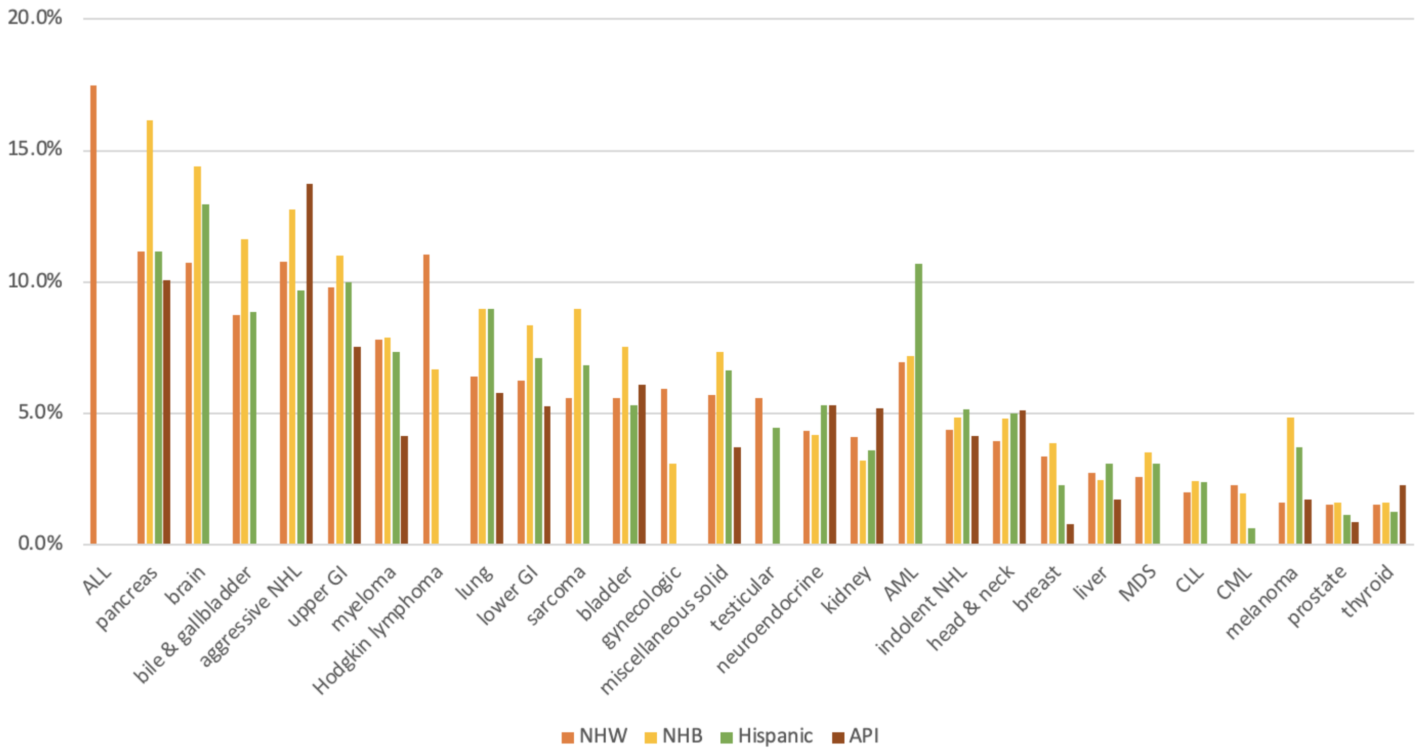

[a]Race/ethnicity groups with fewer than 100 events by specific cancer type were excluded from representation. Abbreviations: ALL, acute lymphocytic leukemia; NHL, non-Hodgkin lymphoma; GI, gastrointestinal; AML, acute myeloid leukemia; MDS, myelodysplastic syndrome; CLL, chronic lymphocytic leukemia; CML, chronic myeloid leukemia; NHW, Non-Hispanic White; NHB, Non-Hispanic Black, API, Asian Pacific Islander

© 2023 Martens KL et al. *JAMA Network Open.*
