# Supplementary material for: Epidemiology of Cancer-Associated Venous Thromboembolism in Patients With Solid and Hematologic Neoplasms in the Veterans Affairs Health Care System
Source: JAMA Netw Open. 2023 Jun 12;6(6):e2317945. doi: 10.1001/jamanetworkopen.2023.17945 (PMC10261992; doi:10.1001/jamanetworkopen.2023.17945)
Supplement: Supplement 2. — Data Sharing Statement [file jamanetwopen-e2317945-s002.pdf]

Data Sharing Statement

Martens. Epidemiology of Cancer-Associated Venous Thromboembolism in Patients With Solid and Hematologic Neoplasms in the Veterans Affairs Health Care System. *JAMA Netw Open*. Published June 12, 2023. doi:10.1001/jamanetworkopen.2023.17945

Data
Data available: No
Additional Information
Explanation for why data not available: VA data underlying this study can be made available to researchers with a VA IRB approved study protocol and data use agreement. Information is available at https://www.virec.research.va.gov or contact the VA Information Resource Center at VIReC@va.gov.
